# Cardiovascular Outcomes in the Patients With Primary Central Nervous System Lymphoma: A Multi-Registry Based Cohort Study of 4,038 Cases

**DOI:** 10.3389/fonc.2021.691038

**Published:** 2021-07-05

**Authors:** Zicong Qiu, Yongshi Tang, Yanting Jiang, Miao Su, Xuemin Wang, Xiuhong Xu, Yuerong Chen

**Affiliations:** ^1^ First School of Clinical Medicine, Guangzhou Medical University, Guangzhou, China; ^2^ Second School of Clinical Medicine, Guangzhou Medical University, Guangzhou, China; ^3^ Sixth School of Clinical Medicine, Guangzhou Medical University, Guangzhou, China; ^4^ Department of Neurology, The First Affiliated Hospital, Sun Yat-Sen University, Guangzhou, China; ^5^ Department of Clinical Medicine, Clinical Medical School, Guangzhou Medical University, Guangzhou, China; ^6^ Department of Acupuncture and Massage Rehabilitation, Integrated Hospital of Traditional Chinese Medicine, Southern Medical University, Guangzhou, China; ^7^ Department of Oncology, Jinshazhou Hospital of Guangzhou University of Chinese Medicine, Guangzhou, China

**Keywords:** primary central nervous system lymphoma, cardiovascular death, SEER database, propensity score matching, risk factor

## Abstract

Primary central nervous system lymphoma (PCNSL) is a rare but highly aggressive non-Hodgkin lymphoma. Treatment-related cardiovascular lesion has become one of the most common complications in patients with tumor. However, very little is known about the cardiovascular death (CVD) of the patients with PCNSL. This study aims at identifying the cardiovascular outcomes of PCNSL patients and making comparison on CVD with extra central nervous system lymphoma (ECNSL). Clinical information of PCNSL and ECNSL was retrieved from the Surveillance, Epidemiology and End Results database. The risk factors of CVD in PCNSL patients and the comparison on the CVD hazard between PCNSL and ECNSL were assessed with the competing risks regression. A 1:2 propensity score matching was used to reduce the imbalanced baseline characteristics between PCNSL and ECNSL. Four thousand thirty-eight PCNSL subjects and 246,760 ECNSL subjects were enrolled in this retrospective study. CVD was the leading cause (41.2%) of non-cancer death in PCNSL patients and mostly occurred within the first year of diagnosis. Age over 60s and diagnosis in 2000–2008 were significantly associated with the elevated risk of CVD in PCNSL patients, while chemotherapy and radiotherapy play no role on the cardiovascular outcomes. Compared with ECNSL patients, the risk of CVD in PCNSL patients were 40% approximately lower. The risk of CVD in the patients with PCNSL still remains unclear currently. Clinicians ought to pay more attention on the risk of CVD in PCNSL patients, especially the elder patients within the first year of diagnosis.

## Introduction

Primary central nervous system lymphoma (PCNSL) is a rare and highly aggressive non-Hodgkin Lymphoma (NHL) that involves the brain, eyes, spinal cord, and cerebrospinal fluid. Although the response rate of PCNSL treatment has been ascending over the past decades, the long-term prognosis of PCNSL patients still remains poor due to the delayed treatment-related complications (1).

Cardiovascular death (CVD) has been regarded as one of the most common late complications of cancer therapy (2, 3). Despite a number of studies demonstrating chemotherapeutic drugs and radioactive exposure are related to the elevation on risk of CVD in the patients with NHL (4–6), research specifically on the cardiovascular outcomes in PCNSL patients has rarely been seen. However, the risk of CVD in PCNSL patients requires our further inspections because of the disparate characteristics of PCNSL differing from other NHL.

Firstly, PCNSL originates from intracranial site with blood brain barrier, leading to the poor access and efficacy in the brain for the chemotherapeutic drugs used in NHL (7, 8). As a result, while patients with NHL are typically treated with rituximab with cyclophosphamide, doxorubicin, vincristine, and prednisolone (R-CHOP) chemotherapy regimen (9), high-dose methotrexate (HD-MTX) based chemotherapy is recommended as the first-line treatment among the individuals with PCNSL (10). In addition, it has been reported that radiation to head and neck was an independent risk factor of ischemic cerebrovascular disease (11–13). What’s more, the median age at diagnosis of PCNSL is 67 years (14), which is older than 54 years in NHL (15), implying that over half of the patients with PCNSL are the elderly and this proportion seems to be enlarging in recent decades (16).

Therefore, considering the disparities in chemotherapy standard, radiotherapy-induced cerebrovascular risk and median age at diagnosis, patients with PCNSL might be suffering from a greater hazard of dismal cardiovascular prognosis.

To date, the clinical outcomes of CVD in the patients with PCNSL still remains unknown. Hence, there is an urgent necessity to differentiate the risk of CVD in the patients with PCNSL from other with NHL, in order to provide more precise references for clinicians on clinical decision making. This study aims to carry out an analysis on cardiovascular outcomes of CVD in the PCNSL patients and its comparison with the extra central nervous system lymphoma (ECNSL).

## Materials and Methods

### Data Source

The cases used in this retrospective cohort study were retrieved from the Surveillance, Epidemiology and End Results (SEER) database, consisting of 18 nationwide cancer registries and covering 35% of the population in the United State (17). All the data were downloaded from the SEER*Stat software (version 8.3.6).

### Study Population

The inclusion criteria were as follows: (1) Year of diagnosis = “2000~2016”; (2) Histological diagnosis = “non-Hodgkin lymphoma”; (3) Type of follow-up = “Active follow-up”; (4) Sequence number = “One primary only”. The exclusion criteria were as follows: (1) Cause of Death and follow-up = “Dead (missing/unknown)”; (2) Race recode = “Unknown”; (3) Marital status at diagnosis = “Unknown”.

### Patient Variables and Outcomes

Subjects were classified into two groups by primary site: PCNSL (Primary Site-labeled = C70.0~C72.9, C75.1, C75.3) and ECNSL (Primary Site-labeled = others). Patient variables comprised age at diagnosis (<60, ≥60), sex (male, female), race (white, black, others), marital status (married, unmarried), histology (defuse large B-cell lymphoma, others), chemotherapy (yes, no/unknown), radiotherapy (yes, no/unknown), surgery (yes, no/unknown), and year of diagnosis (2000–2008, 2009–2016). Subject follow-up outcomes included being alive, cancer-related death, CVD, and other death. As recorded in SEER database, CVD includes disease of heart, hypertension without heart disease, cerebrovascular disease, atherosclerosis, aortic aneurysm and dissection, and other diseases of arteries, arterioles, and capillaries. CVD-specific survival was defined as the period from the date of diagnosis to the patient’s death due to CVD.

### Statistical Analysis

Baseline characteristics were compared with Chi-square Test for the categorical variables. Fine and Gray’s competing-risks regression was used for univariate and multivariate analyses to assess the risk factors of CVD in the patients with PCNSL. Endpoint was defined as CVD and competing event was defined as non-CVD death.

A 1:2 Propensity Score Matching (PSM) which was computed with logistic regression was to reduce the imbalanced baseline characteristics between PCNSL group and ECNSL group. All the variables were enrolled into the propensity score calculation: age at diagnosis, sex, race, marital status, histology, chemotherapy, radiotherapy, surgery, and year of diagnosis. The match was conducted using nearest-neighbor algorithm with caliper width of 0.02. A *P* value over 0.05 was regarded as an acceptable balance. Before and after the PSM, we applied the Fine and Gray’s competing-risks regression to the comparison on the hazard of CVD between PCNSL and ECNSL. The CVD-specific cumulative incidence was displayed by using cumulative curves.

Stata/MP software (version 14.0) was used to perform the competing-risks regression. R software (version 3.6.1) was utilized to carried out the PSM. Hazard ratio (HR) were reported within ninety-five percent confidence interval (95% CI). Difference was defined as statistical significance if a two-tailed *P* value was less than 0.05.

## Results

### Baseline Characteristics Before and After PSM

**Figure 1:**
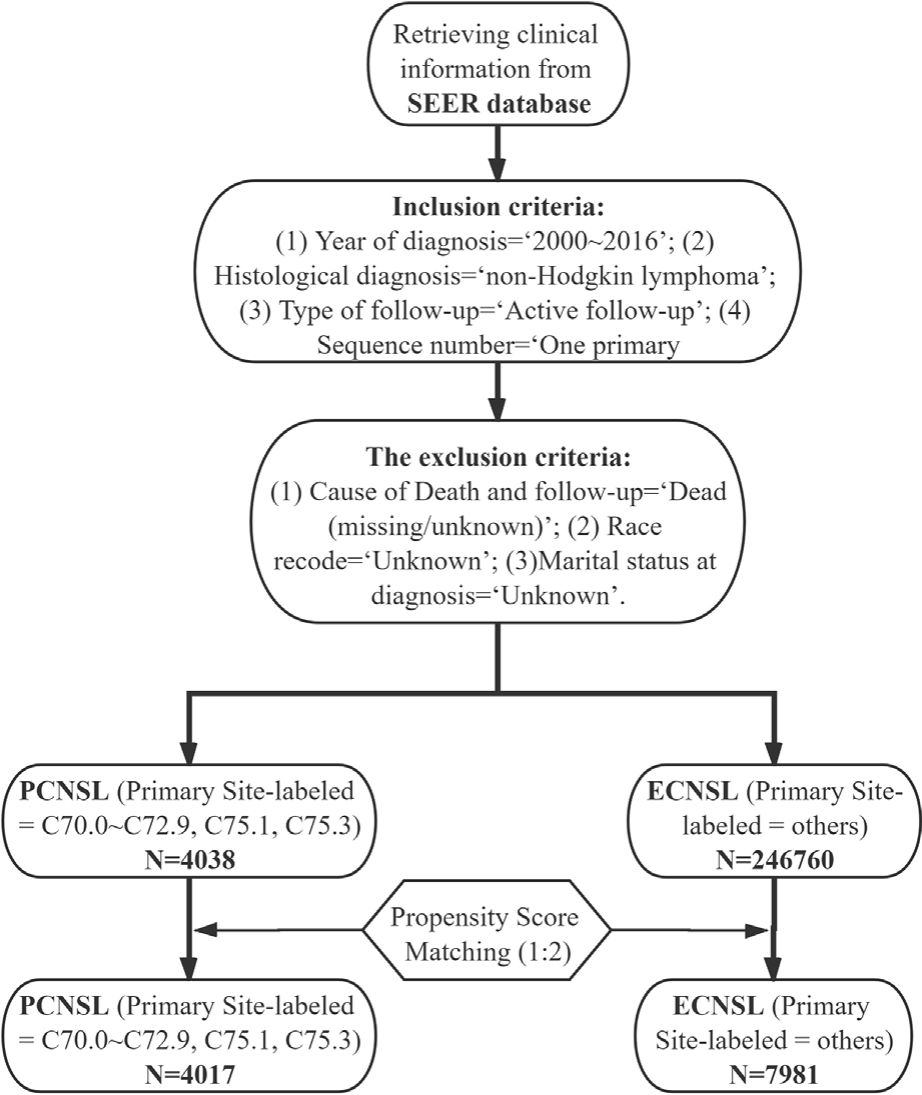
Flowchart outlining the process of inclusion, exclusion, and PSM.

A total number of 2,507,898 cases of NHL were retrieved from the SEER database, consisting of 4,038 cases of PCNSL and 246,760 cases of ECNSL. The median follow-up time was 11 months. Among the patients with PCNSL, age over 60 took up the major part (2,387, 59.1%) and most of them were defuse large B-cell lymphoma (DLBCL) (3,214, 79.6%) and white (3,210, 79.5%). The median age of PCNSL patients was 63 years. As for treatments, while 2,715 (67.2%) patients received chemotherapy, only 1,437 (35.6%) patients received radiotherapy. Over a half (2,304, 57.1%) patients did not underwent surgery. After the 1:2 PSM, all the baseline characteristics were well balanced (*P* > 0.05) between 4,017 cases of PCNSL and 7,981 cases of ECNSL (**Table 1**), (**Figure 1**).

**Table 1:** Baseline characteristics of PCNSL and ECNSL before PSM and after PSM.

Variable	Before PSM (N/%)			After PSM (N/%)		
	PCNSL	ECNSL	*P* value	PCNSL	ECNSL	*P* value
**N**	**4038 (1.6%)**	**246,760 (98.4%)**		**4,017 (33.5%)**	**7,981 (66.5%)**	
**Age at diagnosis**			0.570			0.100
<60	1,651 (40.9%)	99,799 (40.4%)		1,637 (40.8%)	3,128 (39.2%)	
≥60	2,387 (59.1%)	146,961 (59.6%)		2,380 (59.2%)	4,853 (60.8%)	
**Sex**			**0.003**			0.784
Male	2,125 (52.6%)	135,728 (55.0%)		2,112 (52.6%)	4,175 (52.3%)	
Female	1,913 (47.4%)	111,032 (45.0%)		1,905 (47.4%)	3,806 (47.7%)	
**Race**			**<0.001**			0.961
White	3,210 (79.5%)	204,478 (82.9%)		3,207 (79.8%)	6,378 (79.9%)	
Black	345 (8.5%)	25,581 (10.4%)		335 (8.3%)	654 (8.2%)	
Others^#^	483 (12.0%)	16,701 (6.8%)		475 (11.8%)	949 (11.9%)	
**Marital Status**			**0.006**			0.503
Married	2,357 (58.4%)	138,678 (56.2%)		2,351 (58.5%)	4,620 (57.9%)	
Unmarried	1,681 (41.6%)	108,082 (43.8%)		1,666 (41.5%)	3,361 (42.1%)	
**Histology**			**<0.001**			0.849
DLBCL	3,214 (79.6%)	56,589 (22.9%)		3,193 (79.5%)	6,332 (79.3%)	
Others^$^	824 (20.4%)	190,171 (77.1%)		824 (20.5%)	1,649 (20.7%)	
**Chemotherapy**			**<0.001**			0.506
Yes	2,715 (67.2%)	145,937 (59.1%)		2,715 (67.6%)	5,346 (67.0%)	
No/Unknown	1,323 (32.8%)	100,823 (40.9%)		1,302 (32.4%)	2,635 (33.0%)	
**Radiotherapy**			**<0.001**			0.234
Yes	1,437 (35.6%)	39,315 (15.9%)		1,416 (35.3%)	2,726 (34.2%)	
No/Unknown	2,601 (64.4%)	207,445 (84.1%)		2,601 (64.7%)	5,255 (65.8%)	
**Surgery**			**<0.001**			0.993
Yes	1,734 (42.9%)	49,024 (19.9%)		1,718 (42.8%)	3,414 (42.8%)	
No/Unknown	2,304 (57.1%)	197,736 (80.1%)		2,299 (57.2%)	4,567 (57.2%)	
**Year of diagnosis**			**<0.001**			0.228
2000-2008	2,046 (50.7%)	133,840 (54.2%)		2,037 (50.7%)	3,954 (49.5%)	
2009-2016	1,992 (49.3%)	112,920 (45.8%)		1,980 (49.3%)	4,027 (50.5%)	

^#^Other races include American Indian/Alaska Native, Asian/Pacific Islander.

^$^Other histologies include precursor non-Hodgkin lymphoma (B-cell), chronic/small/prolymphocytic/mantle B-cell non-Hodgkin lymphoma, lymphoplasmacytic lymphoma/waldenstrom, Burkitt lymphoma/leukemia, marginal-zone lymphoma, follicular lymphoma, hairy-cell leukemia, plasma cell neoplasms, heavy chain disease, non-Hodgkin lymphoma (B-cell, not otherwise specified), non-Hodgkin lymphoma (T-cell), non-Hodgkin lymphoma (unknown lineage).

PSM, Propensity Score Matching; PCNSL, Primary Central Nerve System Lymphoma; ECNSL, Extra Central Nerve System Lymphoma; DLBCL, Diffuse Large B-Cell Lymphoma. Bold values mean statistical significance (P < 0.05).

### Cause-Specific Mortality

**Figure 2:**
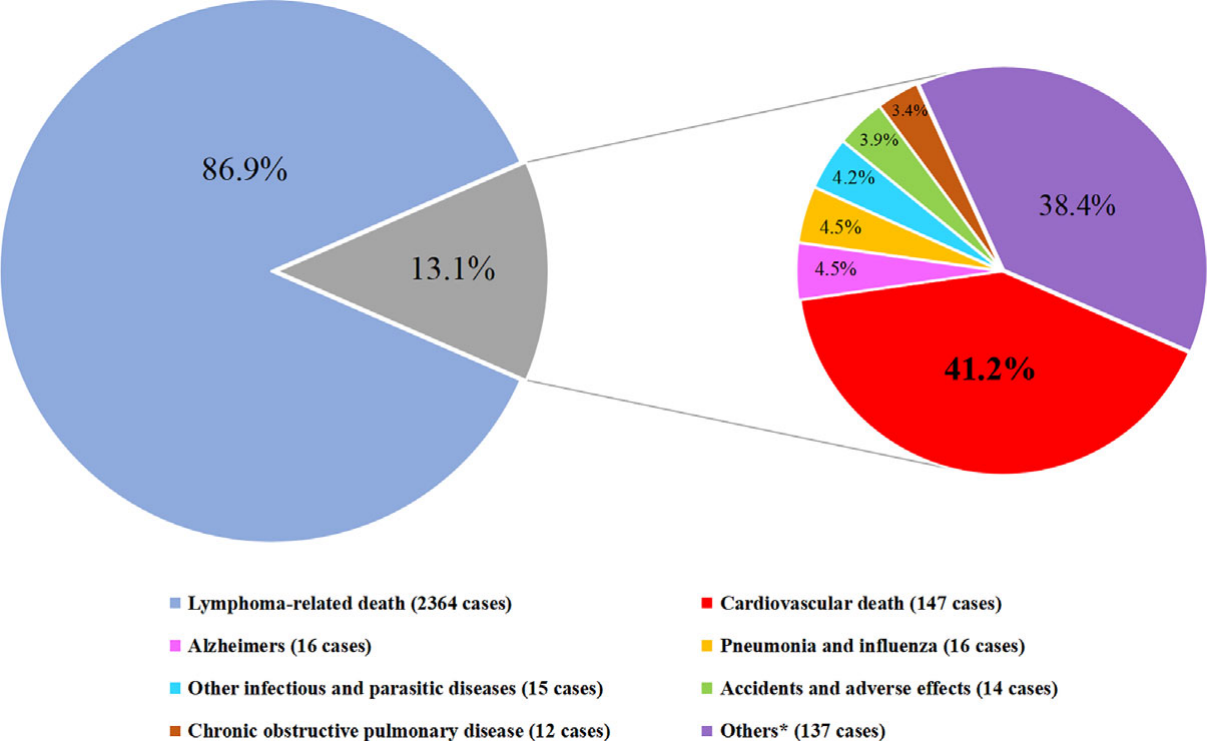
Cause of death distribution in PCNSL patients. (Cardiovascular deaths include diseases of heart, cerebrovascular diseases, hypertension without heart disease, atherosclerosis. Others* include nephritis, nephrotic syndrome and nephrosis, diabetes mellitus, septicemia, chronic liver disease and cirrhosis, stomach and duodenal ulcers, congenital anomalies, symptoms, signs and III-defined conditions, suicide and self-inflicted injury, and other cause of death).

Of those 2,721 PCNSL patients who end with demise, 2,364 (86.9%) were dead due to cancer-related death and the remaining 357 (13.1%) died of non-cancer death, among which CVD accounted for 147 (41.2%) deaths, being the principal cause of non-cancer death (**Figure 2**).

**Figure 3:**
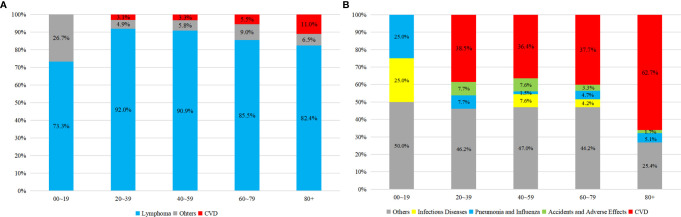
Proportion of causes of death in PCNSLs by different age subgroup. **(A)** all causes of death, **(B)** non-cancer death.

**Figure 4:**
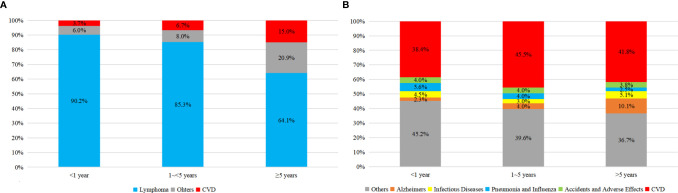
Proportion of causes of death in PCNSLs by different survival time. **(A)** all causes of death, **(B)** non-cancer death.

**Figure 5:**
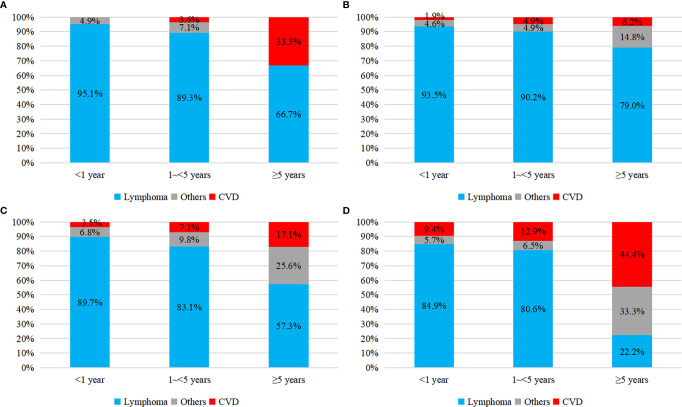
Proportion of all causes of death in PCNSLs by different survival time. **(A)** age at 20~39, **(B)** age at 40~59, **(C)** age at 60~79, **(D)** age at 80+.

As shown in **Figure 3**, there was an obvious growing trend of CVD proportion, from 3.1 to 11.0% among all causes of death and from 38.5 to 62.7% among non-cancer death, as the age of PCNSL patients increasing. Compared to the earlier survival time after the diagnosis of PCNSL, the percentage of CVD was enlarging in latter survival time, from 3.7 to 15% among all causes of death and from 38.4 to 41.8% among non-cancer death, respectively (**Figure 4**). In different age subgroups (20~39, 40~59, 60~79, 80+), all CVDs demonstrated an increasing tendency. Furthermore, in the subgroup of age 80+, the proportion of CVD overtook that of cancer-related death after 5 years follow-up time (**Figure 5**).

Throughout the analysis period, 68 (46.3%) CVD took place within the first year of diagnosis, 46 (31.3%) CVD occurred between the first and the fifth years, and the remaining 33 (22.4%) CVD arose 5 years later. Heart disease was the most frequent (99, 67.3%) CVD among patients with PCNSL and this proportion kept enlarging slightly, followed by cerebrovascular disease (38, 25.9%) and hypertension (9, 6.1%), with only 1 (0.7%) atherosclerosis death (**Table 2**).

**Table 2:** Cardiovascular specific deaths in PCNSL patients by the year after diagnosis.

Cause of death	≤1 year (N, %)	1–5 years (N, %)	≥5 years (N, %)
Heart Disease	44 (64.7%)	31 (67.4%)	24 (72.7%)
Cerebrovascular Diseases	18 (26.5%)	13 (28.3%)	7 (21.2%)
Hypertension without Heart Disease	5 (7.3%)	2 (4.3%)	2 (6.1%)
Atherosclerosis	1 (1.5%)	0 (0.0%)	0 (0.0%)
**Total**	**68 (100%)**	**N = 46 (100%)**	**N = 33 (100%)**

Bold values mean statistical significance (P < 0.05).

### Competing-Risks Regression Analyses of CVD in PCNSL Patients

As shown in **Table 3**, PCNSL patients over 60 were at 2.8-fold higher risk of CVD (HR = 2.833, 95% CI = 1.857–4.323, *P* < 0.001) and those diagnosed in 2009–2016 were related to a lower risk of CVD (HR = 0.592, 95% CI = 0.409–0.858, *P* = 0.006). Specifically, a significant decreased risk of CVD was observed in the patients with chemotherapy (HR = 0.662, 95% CI = 0.464–0.944, *P* = 0.023) in univariate analysis, but not in multivariate analysis (HR = 0.751, 95% CI = 0.525–1.074, *P* = 0.117). Other variables, including marital status, radiotherapy, surgery, histology, race, and sex, played no role on the risk of CVD.

**Table 3:** Competing-risks regression analyses of CVD in PCNSL patients.

Variate	Univariate		Multivariate	
	HR (95% CI)	*P* value	HR (95% CI)	*P* value
**Age at diagnosis**		**<0.001**		**<0.001**
<60	Reference		Reference	
≥60	2.759 (1.810–4.208)		2.833 (1.857–4.323)	
**Sex**		0.463		
Male	Reference		——	
Female	1.137 (0.807–1.603)		——	
**Race**		0.507		
White	Reference		——	
Black	0.635 (0.297–1.356)	0.240	——	
Others^#^	0.935 (0.544–1.605)	0.807	——	
**Marital Status**		0.512		
Unmarried	Reference		——	
Married	0.890 (0.630–1.259)		——	
**Histology**		0.800		
DLBCL	Reference		——	
Others^$^	1.055 (0.699–1.591)		——	
**Chemotherapy**		**0.023**		0.117
Yes	Reference		Reference	
No/Unknown	0.662 (0.464–0.944)		0.751 (0.525–1.074)	
**Radiotherapy**		0.692		
Yes	Reference		——	
No/Unknown	1.073 (0.758–1.519)		——	
**Surgery**		0.563		
Yes	Reference		——	
No/Unknown	0.903 (0.638–1.277)		——	
**Year of diagnosis**		**0.015**		**0.006**
2000–2008	Reference		Reference	
2009–2016	0.634 (0.438–0.916)		0.592 (0.409–0.858)	

^#^Other races include American Indian/Alaska Native, Asian/Pacific Islander.

^$^Other histologies include precursor non-Hodgkin lymphoma (B-cell), chronic/small/prolymphocytic/mantle B-cell non-Hodgkin lymphoma, lymphoplasmacytic lymphoma/waldenstrom, Burkitt lymphoma/leukemia, marginal-zone lymphoma, follicular lymphoma, hairy-cell leukemia, plasma cell neoplasms, heavy chain disease, non-Hodgkin lymphoma (B-cell, not otherwise specified), non-Hodgkin lymphoma (T-cell), non-Hodgkin lymphoma (unknown lineage).

HR, Hazard ratio; CI, Confidence Interval; DLBCL, Diffuse Large B-Cell Lymphoma. Bold values mean statistical significance (P < 0.05).

### Comparative Hazard of CVD Between PCNSL and ECNSL

**Figure 6:**
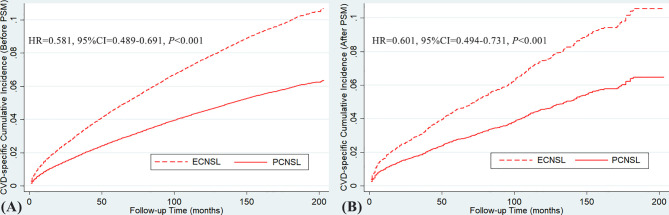
**(A)** CVD-specific cumulative incidence over time in PCNSL and ECNSL before PSM. **(B)** CVD-specific cumulative incidence over time in PCNSL and ECNSL after PSM. (Before PSM: HR = 0.581, 95% CI = 0.489–0.691, *P* < 0.001; after PSM: HR = 0.601, 95% CI = 0.494–0.731, *P* < 0.001).

Before and after the 1:2 PSM, the risk of CVD among PCNSL patients were both approximately 40% lower compared with ECNSL patients (before PSM: HR = 0.581, 95% CI = 0.489–0.691, *P* < 0.001; after PSM: HR = 0.601, 95% CI = 0.494–0.731, *P* < 0.001). As illustrated in **Figure 6**, the cumulative incidence curve of CVD in PCNSL patients was beneath the curve for ECNSL patients, indicating minor incidence of CVD within the analysis period.

## Discussion

In our perspective, this study offers insight into the cardiovascular outcomes in the PCNSL patients.

### Principal Findings

In this multi-registry based cohort study consisting of 4,038 subjects, we found that age over 60 and diagnosed year in 2000–2008 were in correlation with the higher risk of CVD. Among all the causes of non-cancer death in PCNSL patients, CVD was the leading causation, accounting for 147 (41.2%) demises. The majority of CVD occurred within the first year and heart disease was the most frequent CVD. In addition, in the subgroup by no matter age period or survival time, CVD all demonstrated an ascending trend. It is worth noting that in the subgroup of age 80+, the proportion of CVD even exceeded that of cancer-related death after 5 years follow-up time. This result is similar to the previous study. A SEER-based research published on European Heart Journal showed that after 12 years of diagnosis, the cumulative mortality of heart disease even exceeded that of cancer itself in the breast patients with age 75+ (18). Besides, the patients with PCNSL were at a 40% lower risk of CVD compared with those with ECNSL. These results are demanding for more attention to the risk of CVD in the patients with PCNSL, especially in the elder with poor cardiac condition within the first year. With these information, clinical workers can be better aware of cardiovascular risk and consciously detect the early changes in the PCNSL patients’ cardiovascular condition in time, especially those in poor cardiac condition within the first year of diagnosis. Applying these information into clinical practice might greatly reduce the non-cancer death in PCNSL patients and improve the quality of their life.

### Risk Factors of CVD

In the investigation of risk factors, we utilized the competing-risk regression, which is considered as a more suitable and accurate method when estimating the incidence of competing events, rather than using the Kaplan-Meier survival function (19).

#### Age and Year of Diagnosis

The median age of PCNSL patients was 63, which is close to the result of the previous study (20). Our result showed that the patients over 60 were suffering from more than twice as risk of CVD as their counterpart. This result conformed with the findings by Husam et al. who demonstrated that cardiovascular mortality was higher in older breast cancer patients (21). Study from Soisson et al. also showed that endometrial cancer survivors older than 60 years had an increased risk of cardiovascular diseases (22). These results call for the adequate attention to cardiovascular prevention and surveillance for the elder patients. The PCNSL patients diagnosed in latter period (2009–2016) was significantly related to lower risk of CVD compared with previous period (2000–2008) and this might be attributed to the advancement of cardiovascular detection and management over the years (23–25).

#### Chemotherapy

Chemotherapy seems to be a favorable factor of CVD in the univariate analysis, whereas not in the multivariate analysis after enrolling age at diagnosis and year of diagnosis. This inconsistency might be due to the treatment bias on ages: patients younger than 60 were more likely to receive and tolerate the chemotherapeutic regimen because of their better cardiovascular condition, while those over 60 presumably had more contraindications to chemotherapy as a result of dismal cardiovascular comorbidity. Thus, after the adjustment of age of diagnosis, the treatment bias was amended in multivariate analysis.

HD-MTX has been recommended as an indispensable agent in the chemotherapeutic regimen by the clinical guideline (26). Interestingly, some studies indicated that methotrexate was in correlation with reduced cardiovascular risks (27), while some others demonstrated it was not (28). In our study, the cardiovascular risks of chemotherapy in PCNSL patients might be subjected to methotrexate to a large extent, suggesting that methotrexate has no impact on the cardiovascular risks. Nonetheless, it should be notable that the specific chemotherapeutic agent, the dosage, and the courses were not recorded in the database. The effect of methotrexate on the cardiovascular risk still requires more clinical trials.

#### Radiotherapy

In the previous studies, radiation to head and neck was found to be an independent risk factor of ischemic cerebrovascular disease (11–13), whereas the increased cardiovascular risks in the individuals receiving radiotherapy were not observed in our results. This discrepancy might be attributed to the various distribution of radiotherapy dosage across the brain. As reported by some studies (29, 30), only when radiation in the vicinity of the Willis circle arteries will it be a significant risk factor of radiation-associated CVD, otherwise the radiation to other brain structures appeared to play no significant role in the risk of CVD. Similarly, according to N. Haddy et al. (31) studying on the relationship between brain radiation dose and cerebrovascular mortality, the only risk factor was the radiation dose (>30 Gy) received by prepontine cistern, which is an area where Willis circle arteries locates. Nevertheless, information on radiation dosage and method were absent in SEER data and the exact reason of this results still remains unknown.

### Comparison on the Risk of CVD Between PCNSL and ECNSL

After the 1:2 PSM, all the clinical variables, including age, sex, histology, chemotherapy, radiotherapy, and surgery, were well-balanced, which avoided the selection biases as much as possible and allowed the data to be more comparable. The relative risk of CVD in PCNSL patients was 40% lower compared with that in ECNSL patients before and after the PSM. We hypothesized that this disparity might be caused by the different chemotherapeutic drugs for PCNSL and ECNSL. R-CHOP regimen, standard treatment for ECNSL, were confirmed that was significantly associated with elevated risk of congestive heart failure and other cardiovascular diseases (4, 5). Apart from chemotherapy, some confounding variables might potentially also account for the relative risk of CVD, such as cardiovascular comorbidity, diabetes, long-term medicine taking, smoking, alcoholism, etc. Unfortunately, these information were not available in the SEER database and therefore a further analysis was restricted. Such intriguing result calls for more detailed clinical studies.

### Limitations

Several limitations should be noted due to the inherent drawbacks of a retrospective study. The incomplete information of chemotherapy and radiotherapy, such as dosage, drugs, and courses, limited us from a further investigation into these therapeutic risks of CVD. In addition, the SEER database lacked the cardiovascular comorbidity of patients at baseline characteristics, which might result in the potential biases on cardiovascular outcomes. Moreover, we had no access to the profile of other recognized risk factors of CVD, encompassing smoking, hypertension, diabetes, etc. Despite these shortcomings mentioned above, the methods applied in the analyses were rigorously designed and the results still provide certain referential value on cardiovascular outcomes.

### Conclusion

In conclusion, this is a study providing insights into the risk of CVD in the patients with PCNSL. CVD was the leading cause of non-cancer death in PCNSL patients. The proportion of CVD demonstrated an increasing trend among PCNSL patients by different age period and survival time. Thus, clinicians ought to pay more attention on the risk of CVD in PCNSL patients, especially the elder patients within the first year of diagnosis. Chemotherapy and radiotherapy were not relevant with the cardiovascular outcomes of PCNSL patients, which might mitigate the concern of the treatment-induced cardiovascular risk. Compared with ECNSL patients, a more favorable cardiovascular outcome was demonstrated in PCNSL patients. More clinical trials are required to further inspect the risk of CVD in the patients with PCNSL.

## Data Availability Statement

Publicly available datasets were analyzed in this study. This data can be found here: https://seer.cancer.gov/data-software/.

## Ethics Statement

Ethical approval was not required for publicly available information.

## Author Contributions

ZQ, YC, and XX designed the study. ZQ, MS, and XW downloaded, analyzed, and interpreted the data. ZQ, YT, and YJ prepared the draft manuscript. YC and XX provided the critical revisions. All authors contributed to the article and approved the submitted version and agreed with the order of presentation of the authors.

## Funding

This study was funded by the Special Funds for the Cultivation of Guangdong College Students’ Scientific and Technological Innovation. (“Climbing Program” Special Funds) (pdjh2021a0407 and pdjh2021b0416).

## Conflict of Interest

The authors declare that the research was conducted in the absence of any commercial or financial relationships that could be construed as a potential conflict of interest.
