# Supplementary material for: Cardiovascular Outcomes in the Patients With Primary Central Nervous System Lymphoma: A Multi-Registry Based Cohort Study of 4,038 Cases
Source: Front Oncol. 2021 Jul 5;11:691038. doi: 10.3389/fonc.2021.691038 (PMC8289252; doi:10.3389/fonc.2021.691038)
Supplement: Supplementary file 1 [file Table_1.docx]

**Supplementary Table 1. Tumor location and radiotherapy cases of PCNSL and ECNSL.**

| **Tumor location** | **Radiotherapy cases (%)** | **Overall cases (%)** |
| --- | --- | --- |
| **PCNSL** | **1437 (100%)** | **4038 (100%)** |
| Close to WCA* | 23 (1.6%) | 57 (1.4%) |
| Distant from WCA^#^ | 1414 (98.4%) | 3981 (98.6%) |
| **ECNSL** | **41549 (100%)** | **246760 (100%)** |
| Close to heart^$^ | 2234 (5.4%) | 8334 (3.4%) |
| Distant from heart^%^ | 39315 (94.6%) | 238426 (96.6%) |

*Close to WCA was defined as the location including brain stem and pituitary gland.

^#^Distant from WCA was defined as the location including frontal lobe, temporal lobe, parietal lobe, occipital lobe, ventricle (NOS), cranial nerve (NOS), meninges, cerebellum, pineal gland, spinal cord, cauda equina, brain (NOS), central nervous system (NOS).

^$^Close to heart was defined as the location including thoracic esophagus, trachea and bronchus, lung (NOS), intrathoracic lymph nodes, heart, mediastinum, pleura, thymus, breast, thorax (NOS).

^%^Distant from heart was defined as the location including head and neck, upper and lower limbs, abdomen, pelvis and other unspecified site.

PCNSL=Primary Central Nerve System Lymphoma, WCA=Willis Circle Arteries, ECNSL=Extra Central Nerve System Lymphoma, NOS=Not Otherwise Specified.
